# Non-Coding RNAs Potentially Involved in Pyrethroid Resistance of Anopheles funestus Population in Western Kenya

**DOI:** 10.21203/rs.3.rs-3979432/v1

**Published:** 2024-02-29

**Authors:** Isaiah Debrah, Daibin Zhong, Maxwell G. Machani, Godfrey Nattoh, Kevin O. Ochwedo, Collins M. Morang’a, Ming-Chieh Lee, Linda E. Amoah, Andrew K. Githeko, Yaw A. Afrane, Guiyun Yan

**Affiliations:** University of Ghana; University of California, Irvine; Kenya Medical Research Institute; Kenya Medical Research Institute; Sub-Saharan African International Centre of Excellence for Malaria Research, Tom Mboya University; University of Ghana; University of California, Irvine; University of Ghana; Kenya Medical Research Institute; University of Ghana Medical School, University of Ghana; University of California, Irvine

**Keywords:** Anopheles funestus, insecticide resistance, non-coding RNAs, western Kenya, RNA-seq, pyrethroid, PBO, DDT

## Abstract

**Backgrounds:**

The resurgence of *Anopheles funestus*, a dominant vector of human malaria in western Kenya was partly attributed to insecticide resistance. However, evidence on the molecular basis of pyrethroid resistance in western Kenya is limited. Noncoding RNAs (ncRNAs) form a vast class of RNAs that do not code for proteins and are ubiquitous in the insect genome. Here, we demonstrated that multiple ncRNAs could play a potential role in *An. funestus*resistance to pyrethroid in western Kenya.

**Materials and Methods:**

*Anopheles funestus* mosquitoes were sampled by aspiration methods in Bungoma, Teso, Siaya, Port Victoria and Kombewa in western Kenya. The F1 progenies were exposed to deltamethrin (0.05%), permethrin (0.75%), DDT (4%) and pirimiphos-methyl (0.25%) following WHO test guidelines. A synergist assay using piperonyl butoxide (PBO) (4%) was conducted to determine cytochrome P450s’ role in pyrethroid resistance. RNA-seq was conducted on a combined pool of specimens that were resistant and unexposed, and the results were compared with those of the FANG susceptible strain. This approach aimed to uncover the molecular mechanisms underlying pyrethroid resistance.

**Results:**

Pyrethroid resistance was observed in all the sites with an average mortality rate of 57.6%. Port Victoria had the highest level of resistance to permethrin (MR=53%) and deltamethrin (MR=11%) pyrethroids. Teso had the lowest level of resistance to permethrin (MR=70%) and deltamethrin (MR=87%). Resistance to DDT was observed only in Kombewa (MR=89%) and Port Victoria (MR=85%). A full susceptibility to P-methyl (0.25%) was observed in all the sites. PBO synergist assay revealed high susceptibility (>98%) to the pyrethroids in all the sites except for Port Victoria (MR=96%, n=100). Whole transcriptomic analysis showed that most of the gene families associated with pyrethroid resistance comprised non-coding RNAs (67%), followed by imipenemase (10%), cytochrome P450s (6%), cuticular proteins (5%), olfactory proteins (4%), glutathione S-transferases (3%), UDP-glycosyltransferases (2%), ATP-binding cassettes (2%) and carboxylesterases(1%).

**Conclusions:**

This study unveils the molecular basis of insecticide resistance in *An. funestus* in western Kenya, highlighting for the first time the potential role of non-coding RNAs in pyrethroid resistance. Targeting non-coding RNAs for intervention development could help in insecticide resistance management.

## 1.0 Introduction

Vector control particularly the use of bed nets treated with pyrethroids has had an impact on entomological parameters, such as reducing infection rate in the vector population, vector abundance, and parity rate [1, 2] leading to a decline in malaria morbidity and mortality in sub-Saharan Africa as a result of a decline in vectorial capacity [3–5]. Despite these successes, malaria resurgence and outbreaks have been reported in various transmission settings in sub-Saharan Africa where ITNs were deployed [6–9]. Hence, the effectiveness of the primary vector control methods with regard to insecticide resistance needs continuous monitoring and probing of the resistance mechanisms.

In contrast to other major vectors, *Anopheles funestus* sensu stricto (hereafter *An. funestus*) has received very scant attention owing to the difficulty in colonizing this species under laboratory conditions. *An. funestus* is distributed throughout Africa similar to the distributed union of An. gambiae. After developing resistance and exhibiting behavioural adaptability, *An. funestus* has a higher ability to colonize a niche [10, 11]. It is one of the most ubiquitous and efficient malaria vectors in the world; highly susceptible to the P. falciparum parasite, highly anthropophagic and endophilic [12–14]. The significance of studying this mosquito is highlighted by its versatility in ecological adaptation and the emergence of resistance to recommended public health insecticides for vector control [10, 15].

Increased resistance to pyrethroids used for bed net impregnation has led to low efficacy of conventional LLINs against *An. funestus* [16]. Resistance monitoring focuses on transmission foci, hotspots of localized outbreaks, or after spikes in disease cases in pre-elimination and elimination settings [17]. For effective insecticide resistance management, it is essential to genetically characterize insecticide resistance profiles and mechanisms in the vector populations. Metabolic resistance poses the biggest threat to the control of malaria vectors [18]. Cytochrome P450s, Glutathione S-transferases (GSTs) and carboxylesterases (COEs) are well-established enzyme families in malaria vectors known to confer resistance to pyrethroids [19, 20]. These detoxification genes are pivotal in the molecular mechanism of insecticide resistance.

Non-coding RNAs (ncRNAs) form a vast class of RNAs that do not code for protein. Examples of ncRNAs include transfer RNA (tRNA), ribosomal RNA (rRNA), small nuclear RNA (snRNA), small nucleolar RNA (snoRNA), microRNA (miRNA), PIWI-interacting RNA (piRNA), endogenous small interfering RNA (siRNA), circular RNA (circRNA), long non-coding RNA (lncRNA), protein functional effector small ncRNA (pfeRNA), and other ncRNAs whose functions remain unknown [21, 22]. They can control the expression of genes at the chromosomal, transcriptional, post-transcriptional, and translational levels and play a role in the entire developmental process. ncRNAs have been demonstrated in studies on arthropods to be essential for several physiological and developmental processes, including molting, reproduction, immunity, wing development, and insecticide resistance [23]. ncRNAs can modify signalling pathways involved in these biological processes by targeting both DNA and RNA substrates. Sequences of regulatory ncRNAs can also help establish epigenetic alterations such as histone acetylation/deacetylation, DNA/histone methylation, etc. within the nucleus by bringing in chromatin remodelling agents that are known to change transcriptional activity [24, 25]. Based on their length, ncRNAs are arbitrarily divided into two groups: small ncRNAs (scnRNAs, < 200 nts) and long ncRNAs (lncRNAs, > 200 nts)[26]. Depending on where they are in relation to genes that code for proteins, lncRNAs can also be categorized as sense, antisense, intronic, or intergenic [27]. With regards to insecticide resistance in insects, lncRNAs that were found to be differentially expressed during the larval stage development of resistant *Plutella xylostella* genotypes [28] and uniquely differentially expressed during the egg to adult moth stages in Bt-toxin resistant strains of the same insect [29]. Similarly, the expression of the lncRNAs in *P. xylostella* was linked to the expression of the cytochrome P450, the ATP-binding cassette (ABC) transporter and the esterase genes involved in resistance to chlorantraniliprole insecticide [27]. Moreover, some long intergenic non-coding RNAs were overexpressed in deltamethrin-resistant larvae of *Plutella xylostella* exposed to deltamethrin [28]. ncRNAs are intriguing candidates to study when organisms are exposed to insecticides and other toxicants since they are involved in pathways linked to responses to cellular stress [28, 30]. The genes for ribosomal proteins, such as L39 [31], S4 [32], L22 [33], and S29 [34], have been found to be associated with the resistance mechanism of *Culex* mosquitoes.

In the malaria-endemic region of western Kenya, there has been a resurgence of endophilic *An. funestus* and increased 20-fold over a decade ago [9, 35]. The resurgence of this vector was partly attributed to resistance to pyrethroids used in ITN impregnation [36]. As the country is aiming to achieve the malaria elimination goal by 2030, it is very crucial to have a comprehensive understanding of the resistant profile of this important, re-emerged vector to inform stakeholders of the right choice of control strategy to adopt. To date, there have been few investigations on *An. funestus* susceptibility to insecticides in Kenya. The initial study on *An. funestus* susceptibility to insecticides from two study areas in western Kenya was reported in 2007 [37] and even though the species were not identified using molecular techniques, previously identified *Anopheles* species from the same areas revealed that only *An. funestus* was present [38]. Later in western Kenya, seven adults *An. funestus* were sampled and their F1 progenies’ susceptibility to insecticides revealed that they were susceptible to DDT but resistant to permethrin [39]. Further study in Kisumu in the lowland area of western Kenya has shown that *An. funestus* is resistant to pyrethroids (deltamethrin and permethrin) with overexpression CYP6P9a and CYP6P9b responsible for pyrethroid resistance [15]. A recent study in the same Kisumu, using microarray for transcriptome analysis has revealed that overexpression of cytochrome P450s notably, CYP4H18, CYP6M7, CYP9K1, CYP4C36 and CYP4H17 in pyrethroid-resistant *An. funestus* population [40]. The use of microarrays can only be used with the gene families that have been identified on the array, and they only give information on relative expression levels. The RNA-seq technology offers single nucleotide level resolution, absolute rather than relative gene expression profile, and a comprehensive view of the transcriptome in a specific state [41].

In this study, we examined the insecticide resistance profile of *An. funestus* across five sites in four counties in western Kenya and elucidated the molecular mechanisms of resistance using RNA-seq. Our results provide new novel insights into insecticide resistance at the molecular level in this important malaria vector, which has received limited attention, and could help in designing effective control strategies.

## 2.0 Materials and Methods

### 2.1 Sampling of indoor-resting *Anopheles* mosquitoes

**Figure 1:**
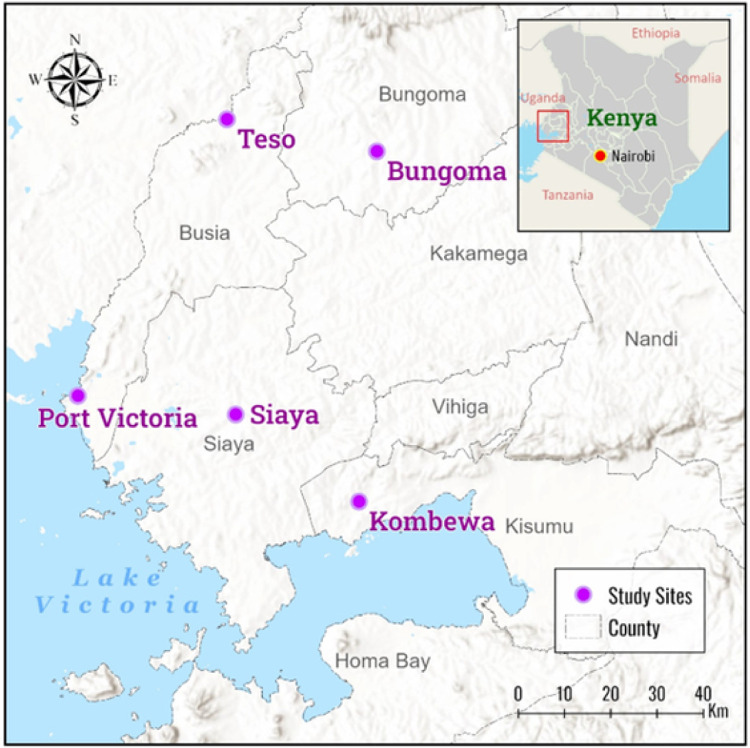
Map of study sites where mosquitoes were sampled in western Kenya. The software ArcGIS Pro 2.6 was used to create the map. Map sources: USGS, ESRI, and CGIAR (www.esri.com)

*Anopheles* mosquitoes were sampled from five sites: Bungoma [00.54057°N, 034.56410°E, 1386–1,545m above sea level (asl)] (highland), Teso (0°43’0” N. 34°21’0” E, 1357–1,500m asl) (highland), Siaya (0.0626° N, 34.2878° E, 1,140–1,400m asl) (lowland), Port Victoria (0° 6’ 0” N / 33° 58’ 0” E, 1,149 asl) (lowland) and Kombewa (0^0^ 07’N, 340 30’E, 1150–1300 m asl) (lowland) in western Kenya (Fig. 1). These sites are malaria-endemic areas predominated by *An. funestus* mosquitoes. Adult *An. funestus* population were sampled from the indoor living room using mouth and prokopack aspiration methods after informed consent was sought and provided by the owners of the households.

### 2.2 Mosquito sorting and identification

Live mosquitoes were sorted by separating male mosquitoes from the females and Culex spp from Anopheles. Later Anopheles mosquitoes were morphologically identified as *An. funestus* s.l and An. gambiae s.l following morphological and taxonomic keys [42, 43].

### 2.3 Raising of F1 progenies.

*An. funestus* blood-fed, gravid and half gravid were put into cages to lay eggs on wet filter papers. The F_0_ females in these physiological states were fed on 10% sugar solutions soaked in cotton wool and laying pads/Petri dishes. After laying the eggs, the eggs were allowed to hatch into larvae. The larvae were put in a pan containing spring water and were fed with the larvae feed, tetramin until they matured to a pupal stage where they were transferred into cages to emerge into adults.

### 2.4 Insecticide susceptibility tests

The F1 adult female mosquitoes aged between 3–5 days old were used for the bioassay. The insecticide susceptibility test was carried out following the standard insecticide tube test method developed by the WHO [17]. The mortality was scored 24 hours post-exposure after maintaining under standard laboratory conditions at a temperature of 27 °C ± 2 °C and a relative humidity of 75% ± 10%.

### 2.5 PBO synergist bioassays

After establishing pyrethroid resistance in the *An. funestus* population, a synergist bioassay was conducted with PBO-impregnated papers to determine the role of P450 monooxygenases in pyrethroid resistance. The PBO inhibits these enzymes’ activity in insects including mosquitoes. The female mosquito samples (F1 progenies) were pre-exposed to 4% PBO for 1 hour before they were immediately exposed to the pyrethroids (0.05% deltamethrin and 0.75% permethrin).

### 2.6 Preparation of samples for molecular and transcriptome analysis

Surviving resistant *An. funestus* samples after exposure to the insecticides (permethrin and deltamethrin) and unexposed (*An. funestus* F1 progenies samples that were not exposed to any insecticides) were killed immediately by keeping them in a deep freezer for about 10 minutes until they were completely knockdown. Samples were immediately stored in 0.5 ml Eppendorf tubes with RNALater and were immediately frozen at −80°C for subsequent molecular and whole transcriptome analysis.

### 2.7 DNA extraction and molecular identification of species

DNA was extracted from the legs of each stored mosquito specimen using the Chelex^®^-100 method [44] and was transferred into pre-labelled 1.5ml storage vials and stored at −20°C for molecular analysis. *An. funestus*-specific PCR was conducted to confirm species using the species-specific primers (ITS2A/FUN) in the internal transcribed spacer region (ITS2) on the ribosomal DNA [45, 46]. Species-specific primers for *An. funestus* (5 − GCATCGATGGGTT ∀TCATG − 3) and universal primer (5 – TGTG ∀CTGCAGGACACAT − 3) were used. A final volume of 12.5 μl of PCR mixture containing 1μl of genomic DNA, 6.5 μl DreamTaq Green PCR Master Mix (2x), 0.5 μl of each of the primers and 4.0 μl of PCR water. Genomic DNA amplification was performed using a T100 thermal cycler (Biorad). The PCR conditions include initial denaturation at 95 °C for 3 seconds, denaturation of 94 °C for 30 seconds, annealing at 55 °C for 30 seconds for 34 cycles, extension at 72 °C for 45 seconds and final extension at 72 °C for 6 seconds. The DNA bands were visualized using the agarose gel electrophoresis.

### 2.8 RNA extraction

Total RNA was extracted from a pool of ten mosquitoes for each group (pyrethroids-resistant group and unexposed to pyrethroids). Total RNA was isolated and purified from the whole mosquito using the ZYMO Quick-RNA miniprep kit [47]. The details of the pooled samples is shown in Table 1.

### 2.9 cDNA library preparation and RNA Sequencing

The quality of each total RNA sample was assessed using High Sensitivity RNA Tapestation (Agilent Technologies Inc., California, USA) and quantified with Qubit 3.0 RNA HS assay (ThermoFisher, Massachusetts, USA). Ribosomal RNA was depleted with Ribo-Zero Plus rRNA Removal Kit (Illumina Inc., California, USA). Samples were then heated, fragmented and randomly primed according to the manufacturer’s recommendation. The first strand was synthesized with the Protoscript II Reverse Transcriptase with a longer extension period, approximately 30 minutes at 42 C. All remaining steps for library construction were performed according to the NEBNext^®^ Ultra^™^ II Directional RNA Library Prep Kit for Illumina^®^ (New England BioLabs Inc., Massachusetts, USA). Final libraries quantity was assessed by Qubit 3.0 (ThermoFisher, Massachusetts, USA) and quality was assessed by TapeStation D1000 ScreenTape (Agilent Technologies Inc., California, USA). The final library size was about 350bp with an insert size of about 200bp. Illumina^®^ 8-nt dual indices were used. Equimolar pooling of libraries was performed based on QC values and sequenced on an Illumina^®^ NovaSeq platform (Illumina, California, USA) with a read length configuration of 150 PE for [120M PE] reads per sample (60M in each direction).

### 2.10 Bioassay data analysis

The mortality rate of the sample tested was expressed as the total number of dead *An. funestus* mosquitoes of all the replicates exposed to a particular insecticide and expressed this as the percentage of all the population exposed to that insecticide. Abbott’s formula was used to correct mortality if the mortality at 24 hours in the control tube was between 5% and 20%. Following the WHO criteria [17] for determining insecticide resistance in the malaria vector population, a population is classified as susceptible when the mortality is between 98–100%, resistant when mortality is less than 90% and suspected resistance when mortality is between 90–97%.

### 2.11 Quality control and differential expression analysis

Upon obtaining paired-end sequence reads from the sequencing centre (range 46,981,760–96640964 total reads), they were checked for quality using *FASTQC* (v0.11.5) [48] and cleaned to remove adapters (Table 1). Trimmomatic module (v.0.39) [49] was used to remove the Illumina adapters (TruSeq3-PE-2) that were used to construct the library, which resulted in the selection of reads that were more than 50 bp and a Phred-Quality-Score greater than 20 for downstream analysis. The resulting reads were confirmed to be of acceptable quality by running *FASTQC* (v0.11.3) before they were aligned to the reference genome. The *Anopheles funestus* FUMOZ genome from VectorBase (AfunF3.53) was used as the reference genome and this was aligned using the HISAT2 v2.2.1, which involved building the HISAT2 index file for the genome [50]. To reduce the size of the output SAM tools from the alignment output, they were converted to BAM files, sorted, and indexed using SAMtools v1.10 (Li *et al*., 2009). The sorted and indexed files were used as input for the Htseq-count reads, which were created using the module htseq-count (v.0.6.1) as described [52]. A reference gene transfer format was used to count the number of alignment mapping to each gene based on union and intersection-strict [52]. Gene expression values were normalized using Relative Log Expression (RLE) from DESeq2. Expression abundance between different treatments (pyrethroids-resistant group vs pyrethroid susceptible FANG colony of *An. funestus* mosquitoes [53] and unexposed vs the susceptible FANG colony) for the study sites (Teso, Port Victoria, Siaya and Kombewa) was determined using DESeq2 (v.1.18.0) [54]. The susceptible FANG colony raw sequence data was retrieved from the GenBank (accession: ERR981209–ERR981211). A correlation of gene expression between biological replicates was calculated by Pearson’s correlation as suggested before [55], while the Benjamini-Hochberg method was applied in calibrating the p-value to decrease chances of false positives [56]. Therefore, differential expression between treatment groups was considered significant if the p-value < 0.05 and the fold change (FC) > 1.5 [57]. Hierarchical clustering analysis was applied to cluster genes exhibiting similar expression patterns/levels, while the Gene Ontology (GO) from the GO database (http://geneontology.org/) was utilized for functional analysis of the differentially expressed genes to establish their biological profiles (52).

## 3.0 Results

### 3.1 Phenotypic resistance profile in western Kenya

Pyrethroid resistance was observed in all the sites with an average mortality rate (MR) of 57.6%. Port Victoria had the highest level of resistance to permethrin (MR = 53%) and deltamethrin (MR = 11%) pyrethroids. Teso had the lowest level of resistance to permethrin (MR = 70%) and deltamethrin (MR = 87%). Resistance to DDT was observed only in Kombewa (MR = 89%) and Port Victoria (MR = 85%). However, after samples were pre-exposed to the synergist, PBO, high susceptibility (> 98%) to the pyrethroids (deltamethrin and permethrin) was observed in all the sites except for Port Victoria where suspected resistance (96%) was observed for PBO + deltamethrin. In addition to the pyrethroid resistance, resistance to DDT was observed in Kombewa (89%) and Port Victoria (85%) (Table 2). Notwithstanding, a suspected resistance to DDT was observed in Siaya (93%) and Teso (92%). *An. funestus* was, however, fully susceptible to pirimiphos- methyl (0.25%) in all the sites.

### 3.2 Differentially expressed genes between groups.

**Figure 2:**
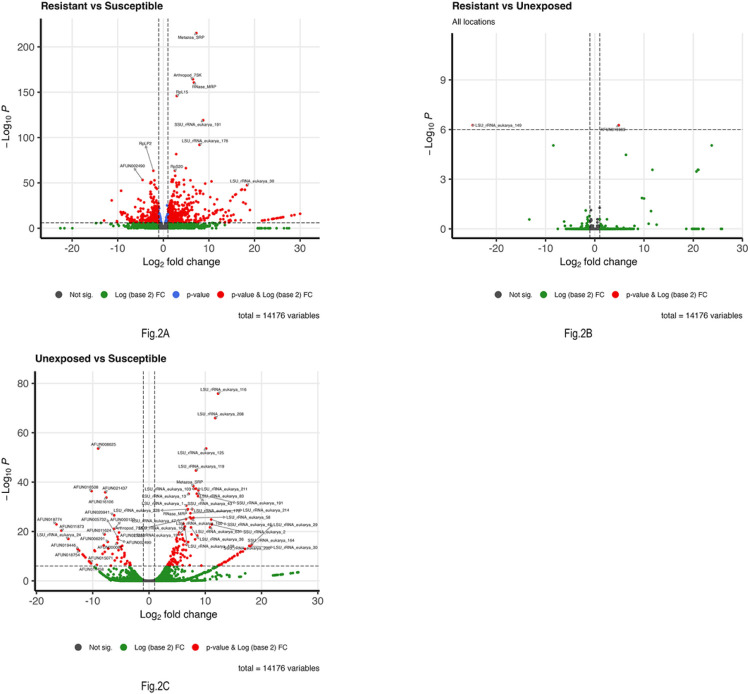
Volcano plot indicating upregulation and downregulation for resistant vs susceptible (A), resistant vs unexposed (B) and unexposed (control) vs susceptible (C). The X-axis indicates the log2 fold-change- positive and negative values are up and down-regulated respectively relative to the susceptible group in A and C. The Y-axis indicates −log_10_ of the adjusted P-value (FDR) (−log_10_FDR values >200 for A, > 9 for B and > 80 for C). In each volcano plot, genes that are overexpressed in the population are >0 on the x-axis. P-values of < 0.05 are indicated by the horizontal line, while 2-fold expression differences are indicated by vertical dotted lines.

After quality control and the elimination of genes with low read counts, differential expression analysis was carried out on the transcripts. Three pairwise comparisons were performed: resistant versus susceptible, resistant versus unexposed (control) and unexposed versus susceptible. The resistant versus unexposed comparison helps to account for the induction of transcription during the pyrethroid exposure; genes were filtered by analyzing their expression profiles in the susceptible *An. funestus* population, under the assumption that constitutive resistance genes will be significantly differentially expressed between both survivors of the bioassay and the unexposed field F1 progenies when compared to the susceptible FANG colony. The volcano plots (Fig. 2) showed the downregulation and upregulation of the genes between resistant vs susceptible (Fig. 2A), resistant vs unexposed (Fig. 2B) and unexposed vs susceptible (Fig. 2C). There was a clear distinction between overexpression of genes in resistant vs susceptible (Fig. 2A) and unexposed vs susceptible (Fig. 2C) group comparisons. However, there was no difference in the gene overexpression between the resistant and unexposed group comparisons.

**Figure 3:**
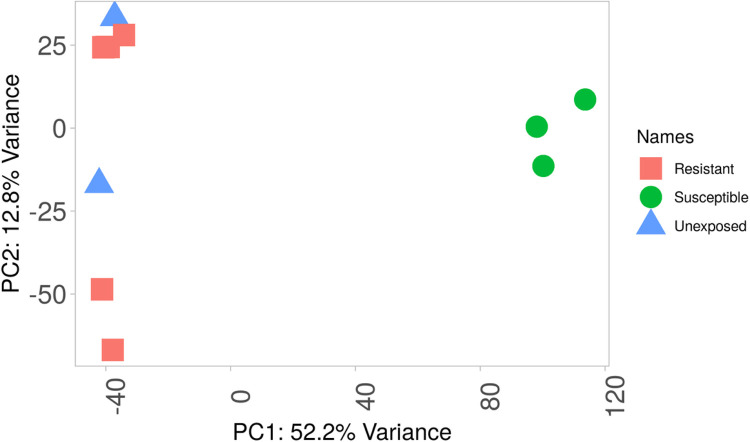
A principal component analysis showing the gene expression pattern of the sample groups relative to the susceptible group.

To summarize the expression pattern of genes in the sample groups, principal component analysis (PCA) and heatmap were used. The PCA plots indicate a representation of differences in the sample groups (resistant, unexposed and susceptible). The samples in the resistant and unexposed groups clustered together to the left-hand side away from the susceptible counterparts indicating the similarity between them (Fig. 3). The susceptible FANG group clusters towards the left side away from the resistant and the unexposed groups (Fig. 3).

**Figure 4:**
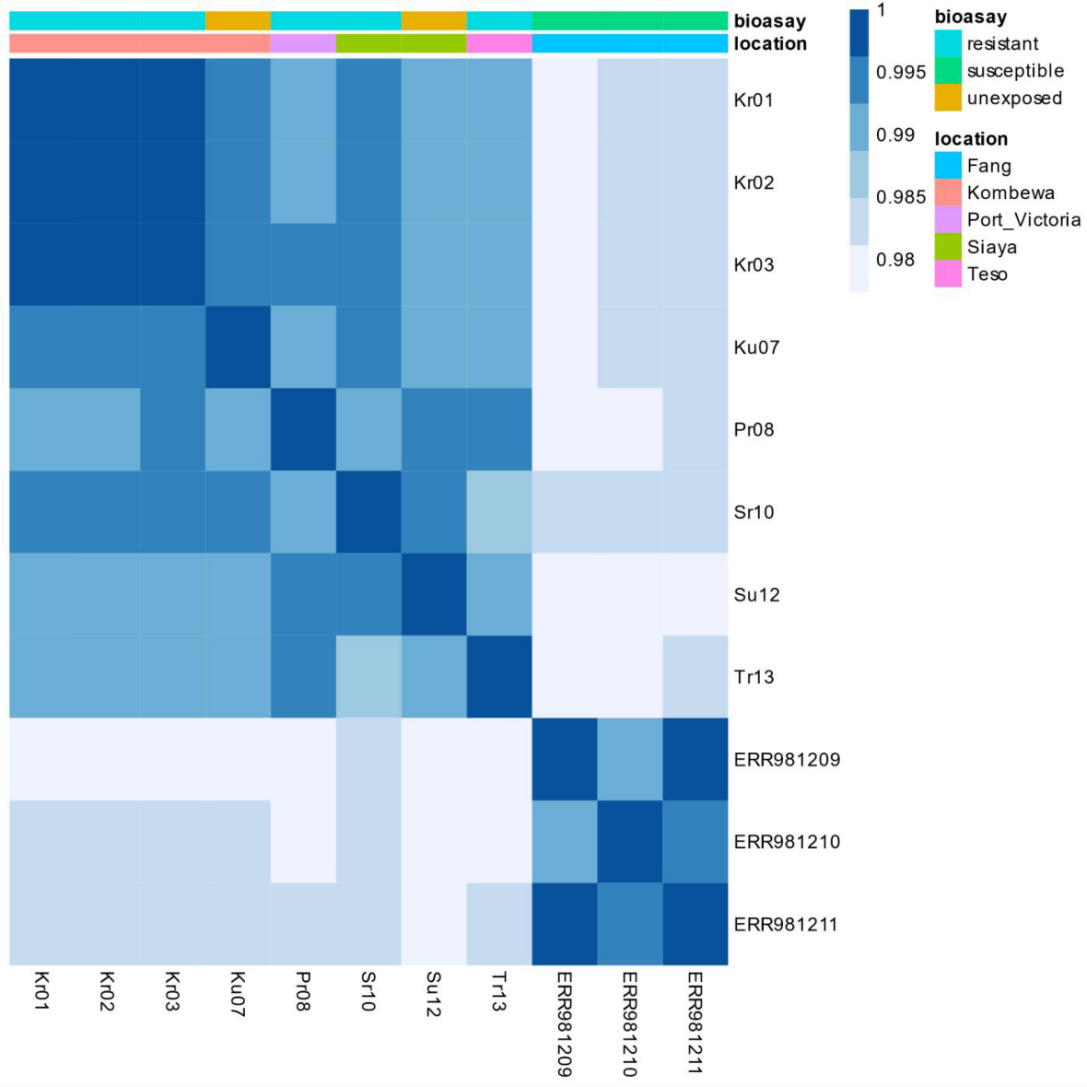
Heatmap indicating the expression of genes in the sample groups relative to the susceptible group.

The heatmap revealed that there was an obvious grouping of the samples into resistant, unexposed and susceptible. Dissimilarity in the gene expression levels was noticed between the groups. The overall gene expression profile indicates a higher level of expression in the resistant and unexposed sample groups compared to the susceptible (Fig. 4). Moreover, most of the genes were highly expressed in the Kombewa-resistant (Kr01, Kr02 and Kr03) samples. This was followed by the resistant samples from Port Victoria, Siaya and Teso. However, low levels of gene expression were observed in the susceptible samples (ERR981209, ERR981210 and ERR981211).

**Figure 5:**
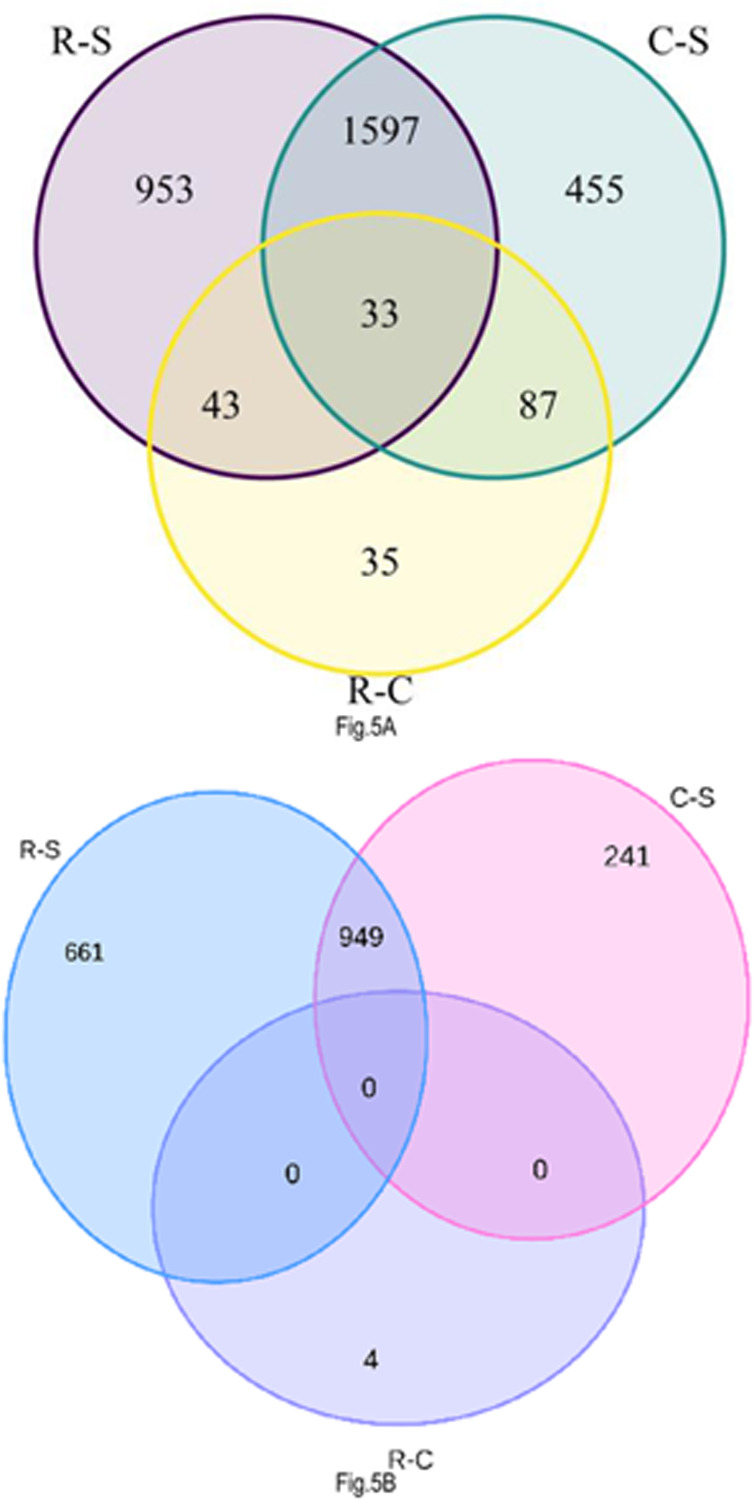
Venn diagram comparing upregulated and downregulated genes between-group comparisons. A indicates upregulated genes between the groups and B indicates downregulated genes between groups. R-S: field-resistant population that survived pyrethroid exposure vs susceptible colony, R-C: field-resistant population that survived pyrethroid exposure vs unexposed (control) field population and C-S: unexposed (control) field population vs susceptible colony.

Comparison using the Venn diagrams, 33 genes (n = 14176) were differentially expressed in all the comparisons [resistant vs susceptible (R-S), resistant vs unexposed (R-C) and vs unexposed and susceptible (C-S)] (Fig. 5A). However, 953, 35 and 455 common genes were differentially expressed in only R-S, R-C and C-S respectively. More genes (1597) were significantly differentially expressed between R-S and C-S comparisons compared to the other comparisons. This was followed by 87 differentially expressed genes observed between C-S and R-C comparisons and 43 differentially expressed genes between R-S and R-C comparisons (Fig. 5A). Most of the downregulated genes were found in the R-S and this was followed by C-S (Fig. 5B). Nine Hundred and forty-nine (949) genes were downregulated between R-S and C-S comparisons (Fig. 5B).

### 3.3 Differentially expressed ncRNAs linked to pyrethroid Resistance.

**Figure 6:**
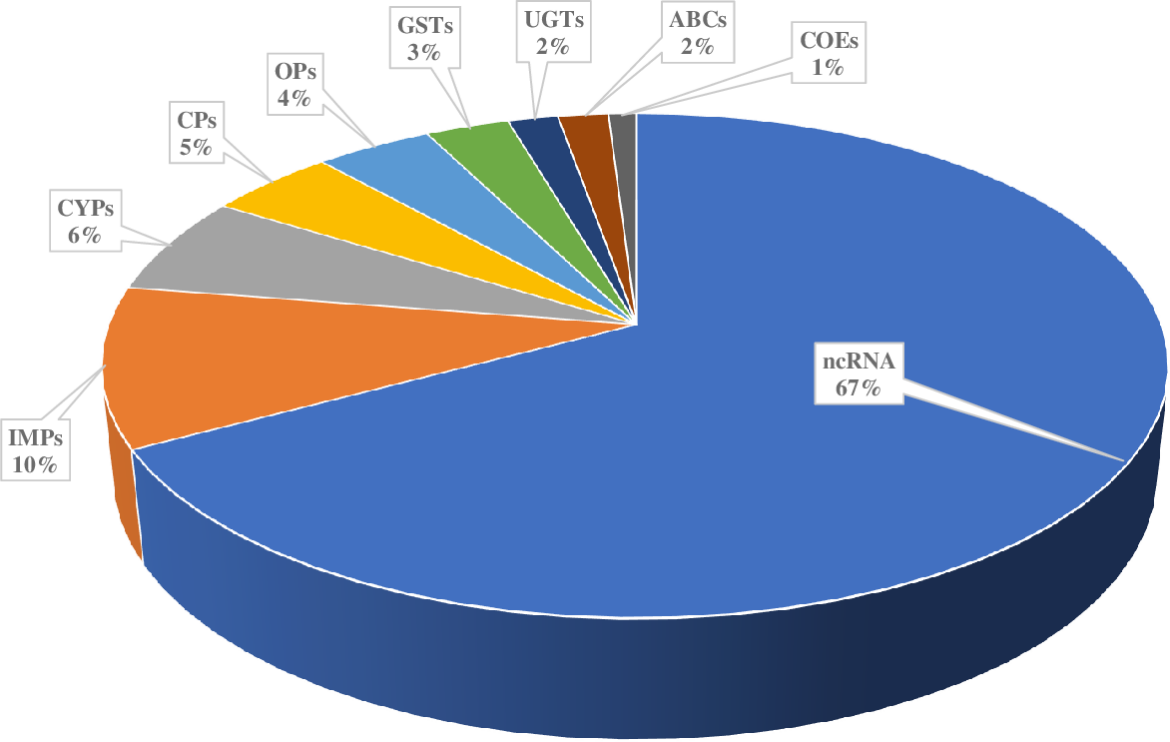
Pie chart showing the proportion of gene family involving pyrethroid resistance. **IMPs:** Imipenemase, **CYPs:** Cytochrome P450s, **CPs:** cuticular proteins, **OPs:** olfactory proteins, **GSTs:** Glutathione S-transferases, **UGTs:** UDP-glycosyltransferases, **ABCs:**ATP-binding cassettes, **COEs:** carboxylesterases and **ncRNA:** non-coding RNA

Differentially expressed ncRNAs between resistant vs susceptible (R-S) and unexposed vs susceptible (C-S) were determined by a fold change FC > 1.5 and FDR < 0.05. The whole transcriptome analysis shows that ncRNAs constituted 67%, the highest proportion of the gene families involved in pyrethroid resistance (Fig. 6). This was followed by IMPs (10%), CYPs (6%), CPs (5%), OPs (4%), GSTs (3%), UGTs (2%), ABCs (2%) and COEs (1%) (Fig. 6).

The main ncRNAs that were overexpressed are in the resistant vs susceptible (R-S) and unexposed vs susceptible (C-S) are Metazoa_SR, RNaseP_nu, U3_1, Arthropod_7S, LSU_rRNA_eukarya_, SSU_rRNA_eukarya_2, LSU_rRNA_eukarya_13, SSU_rRNA_eukarya_46, LSU_rRNA_eukarya_2, LSU_rRNA_eukarya_3, SSU_rRNA_eukarya_15, LSU_rRNA_eukarya_5, LSU_rRNA_eukarya_6, SSU_rRNA_eukarya_164, SSU_rRNA_eukarya_19, SSU_rRNA_eukarya_200, LSU_rRNA_eukarya_155, LSU_rRNA_eukarya_17, LSU_rRNA_eukarya_17, LSU_rRNA_eukarya_214 and RNase_MRP (Table 3).

### 3.4 Differentially expressed metabolic genes associated with pyrethroid resistance.

Similarly, to identify the main genes in the enzyme families responsible for high pyrethroid metabolic resistance, FDR < 0.05 and a fold change FC > 1.5 were used. The main enzyme families identified are the cytochrome P450s, GSTs, salivary gland proteins, Peptidase S1 domain-containing proteins, UGTs and sulfotransferases (Table 4). However, most of these genes were moderately differentially expressed. The findings indicate that in western Kenya, different genes within these enzyme families were responsible for resistance (Table 4). The top cytochrome P450 enzymes are moderately overexpressed in the *An. funestus* in western Kenya were CYP6P9, CYP6P9, CYP6N, CYP6N, CYP9J, CYP49A, CYP6P, AFUN02089, AFUN01936, CYP9K, CYP304B. These genes were overexpressed in resistant vs susceptible and unexposed/control vs susceptible group comparisons. However, CYP304C1 and CYP315A1 were overexpressed only in the resistant vs susceptible comparison (Table 4). Among the GSTs, the overexpressed genes in resistant vs susceptible and unexposed/control vs susceptible groups are GSTD, GSTT, GSTE, GSTD, and GSTD3 were overexpressed only in the resistant vs susceptible comparison (Table 4). AFUN02142, AFUN021428 and AFUN019106 were the only cuticular proteins that were overexpressed in the resistant vs susceptible comparison. The differential expression analyses revealed that some of these UGTs were overexpressed in the resistant vs susceptible and unexposed/control vs susceptible groups comparisons (Table 4). These include UGT302A, UGT310B, UGT308D, UGT306A3 and AFUN003620. AFUN016205 and AFUN016207 were the sulfotransferases that were overexpressed in the *An. funestus* population from western Kenya (Table 4). The summary of the RNA-seq data set for the FC and P-values of each gene is presented in Additional file 1.

### 3.5 Gene ontology analysis of the differentially expressed genes

**Figure 7:**
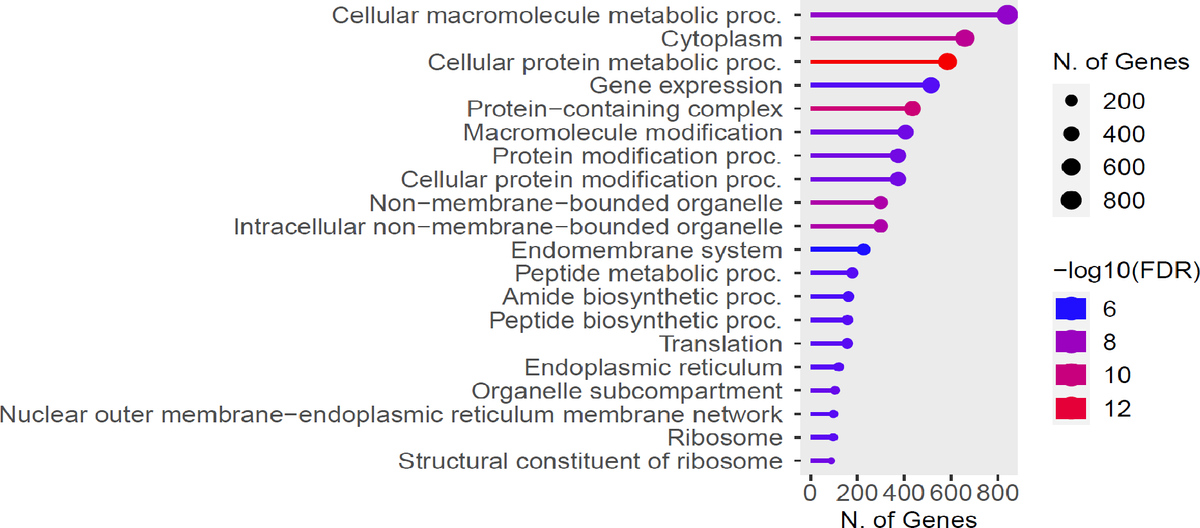
Gene Ontology (GO) enrichment analysis of the differentially expressed genes. The x-axis indicates the gene count/number of genes while the y-axis indicates the enriched terms. The colour is used to distinguish at different levels.

GO term annotation pathways analysis was employed to elucidate the biological functions and signalling pathways that may be regulated by the differentially expressed genes in *An. funestus*. Our findings revealed that these genes were engaged in a wide variety of biological functions and signalling pathways. Detailed GO enrichment for the differentially expressed genes in ontologies of cellular components, biological processes, and molecular function is represented in Fig. 7. By GO annotation, the differentially expressed genes in pyrethroid-resistant *An. funestus* were enriched mostly in cellular macromolecule metabolic processes, cytoplasm, cellular protein metabolic processes and gene expression (Fig. 7).

## 4.0 Discussion

The successful implementation and development of insecticide resistance management measures depends on elucidating the mechanisms underlying resistance in malaria vectors. In this study, we have characterized the phenotypic resistance profile of *An. funestus* and the molecular basis of pyrethroid resistance in western Kenya. This is one of the most comprehensive studies on the *An. funestus* susceptibility status to pyrethroids and DDT in western Kenya.

Our study revealed a high level of pyrethroid resistance across western Kenya although resistance levels vary from site to site. In addition, resistance to DDT has been detected in Kombewa and Port Victoria. This confirmed a previous study in East Africa including western Kenya which reported widespread pyrethroid resistance in the *An. funestus* population [15]. The rise of multiple resistance of *An. funestus* was also confirmed in a previous study in western Kenya [15], Benin, west Africa [59] and Malawi, southern Africa [60]. *An. funestus* was, however, fully susceptible to pirimiphos methyl, the organophosphate in all the study sites. This is congruent with a previous study in Tanzania where a full susceptibility of this vector to pirimiphos methyl in Tanzania was reported [12]. This is an indication that this insecticide can still be maintained for IRS programs in western Kenya. The preexposure of samples to the synergist, PBO has shown that *An. funestus* was fully susceptible to the pyrethroids in all the study sites except Port Victoria where 96% mortality was observed for the PBO + deltamethrin. This implies that the metabolic resistance mechanism (cytochrome P450 monoxygenases) was fully involved in insecticide resistance in the *An. funestus* in these sites but partially involved in Port Victoria [17]. Other mechanism(s) might be contributing to pyrethroid resistance in Port Victoria leading to that site having the highest level of resistance compared to the other sites.

*An. funestus* has no kdr markers for resistance [61] hence metabolic resistance mechanism through overexpression of detoxification genes plays a crucial role in insecticide resistance [62, 63]. In this study, we have identified the top twenty ncRNAs that were differentially expressed in resistant and unexposed field populations of *An. funestus* from western Kenya. Although their mechanisms of pyrethroid resistance in *An. funestus* is unknown, they could be playing a role in regulating the expression of pyrethroid-resistant metabolic genes in the *An. funestus* resistant populations. Our findings add up to a body of evidence which hypothesised that ncRNAs play roles in insecticide resistance development [28]. In general, the biological roles of ncRNAs in detoxification and insecticide resistance pathways are poorly understood. However, few studies have reported that some ncRNAs (notably microRNAs) interfered with the expression of insecticide-detoxifying enzymes. For instance, MiR-2b-3p has been proposed to potentially suppress the cytochrome P450 9f2 (CYP9F2) gene’s transcriptional activity, which would impede the larvae of *P. xylostella* from progressing through developmental detoxification pathways [64]. Furthermore, it has been observed that an overabundance of miR-13664 reduced the cytochrome P450 314A1 (CpCYP314A1) gene’s mRNA expression levels, increasing *Culex pipiens pallens* (Diptera: Culicidae) susceptibility to deltamethrin [65]. Given that a few proportions of the metabolic gene families (IMP, cytochrome P450, CP, OP, GST, UGTs, ABCs and COE.) were identified in this study to be involved in pyrethroid resistance and were mostly moderately overexpressed, this large proportion of highly overexpressed ncRNAs may play a crucial role in regulating their expression.

Studies have established those genes belonging to the esterase, cytochrome P450, esterases, GSTs, UGTs, cuticular proteins and ABC transporter families are implicated in insecticide resistance [19, 40, 66–68]; as a result, ncRNAs could be considered for designing RNAi-based control systems. This will, however, require a deeper understanding of the molecular mechanisms underlying RNAi-based control systems in mosquitoes since there already existing gaps in understanding this technology in controlling other insects [69]. Furthermore, high-throughput sequencing techniques have recently yielded important new information about the functions of ncRNAs in insect development and the evolution of insecticide resistance [69]. Non-coding rRNAs constitute over 80% of the total cellular RNA in mosquitoes [70]. In this study, the majority of rRNA was removed using the RiboZero Plus kit. Typically, the literature indicates that rRNA comprises anywhere from 1 to 20% of the final rRNA-depleted sequencing libraries [71]. Our results demonstrate that our RNA-seq method can effectively detect and quantify both coding and non-coding RNA.

Two different ncRNA-based insect management approaches have been proposed following these findings: (a) using biodegradable ncRNA-insecticide solutions to control insects, [72, 73] and (b) using metabolic engineering techniques to find and take advantage of target species’ ncRNA-associated signalling pathways [74]. However, the molecular mechanisms behind the functioning of ncRNAs in detoxification and insecticide resistance signalling pathways are still not clear, despite the mounting body of evidence suggesting these molecules are significant regulators of insect development [28, 69, 75]. This is because research in this area is still in its early stages. The straightforward CRISPR-Cas9 genome editing technique has the potential to generate novel understandings of the roles of regulatory ncRNA sequences as well as ncRNA-based techniques targeted at managing insects including disease vectors. Moreover, using inhibitors to target specific ncRNAs might interfere with the expression levels and reduce or reverse insecticide resistance. Thus, ncRNAs could be potential targets for vector control in the future. Recently, Oberemok *et al*. [76] proposed an innovative strategy to tackle insecticide resistance and create safer compounds. Their method employs synthetic DNA oligomers to disrupt gene expression by targeting ribosomal RNA (rRNA) rather than messenger RNA (mRNA). Because rRNA makes up 80% of cellular RNA and is more plentiful and stable than mRNA, it represents a promising target for DNA antisense oligonucleotide (ASO) interventions. This strategy seeks to offer a more efficient and enduring approach to combating insecticide resistance.

*An. funestus* is a notorious vector of human malaria in Africa and has contributed to over 90% of all malaria transmission in some parts of eastern and southern African regions [12, 13]. It is noteworthy that the outcome of our study represents an advancement in the molecular basis of insecticide resistance in *An. funestus* population. Our comprehension of the functional importance of ncRNAs in insecticide resistance pathways could enable the creation of ncRNA-based vector control techniques to control *An. funestus*.

## Conclusions

*An. funestus* population is highly resistant to pyrethroids in western Kenya with Port Victoria recording the highest levels of resistance to the type I and type II pyrethroids. However, preexposure to PBO synergists recorded high susceptibility to the pyrethroids except in Port Victoria. We have shown for the first time that insecticide resistance in *An. funestus* is linked to the expression of ncRNAs hence a better understanding of these molecular events could help to develop resistance management strategies for future malaria control.

## Figures and Tables

**Table 1:** RNA-seq read filtering and mapping statistics.

Sample ID	Site	Phenotype	Pool	Raw reads	Clean reads	Mapped reads	% Mapped
Kr01	Kombewa	Resistant	10 mosquitoes	69034333	68982973	68982973	100%
Kr02	Kombewa	Resistant	10 mosquitoes	66278265	66237148	66237148	100%
Kr03	Kombewa	Resistant	10 mosquitoes	82298618	82251274	82251274	100%
Ku07	Kombewa	Unexposed	10 mosquitoes	68512143	68463374	68463374	100%
Pr08	Port Victoria	Resistant	10 mosquitoes	81542127	81505195	81505195	100%
Sr10	Siaya	Resistant	10 mosquitoes	96640964	96603256	96603256	100%
Su12	Siaya	Unexposed	10 mosquitoes	72322576	72294283	72294283	100%
Tr13	Teso	Resistant	10 mosquitoes	92317683	92276651	92276651	100%
ERR981209	FANG colony	Susceptible		61161962	60881678	60881678	100%
ERR981210	FANG colony	Susceptible		44384316	44042916	44042916	100%
ERR981211	FANG colony	Susceptible		55329478	55063614	55063614	100%

**Table 2:** Mortality rate of *An. funestus* exposed to different insecticides and synergists (24-hr post-exposure).

Study site	Type and % of insecticide/chemical used	N	% Mortality (24hr)
Kombewa	Permethrin (0.75%)	280	54
	Permethrin (0.75%) + PBO (4%)	300	99
	Deltamethrin (0.05%)	180	59
	Deltamethrin (0.05%) + PBO (4%)	100	100
	DDT (4%)	100	89
	Pirimiphos methyl (0.25%)	180	99
Siaya	Permethrin (0.75%)	100	78
	Deltamethrin (0.05%)	133	52
	0.75% permethrin + PBO (4%)	100	100
	Deltamethrin (0.05%) + PBO (4%)	100	100
	DDT (4%)	100	93
	Pirimiphos methyl (0.25%)	100	100
Teso	Deltamethrin (0.05%)	100	70
	Permethrin (0.75%)	100	87
	0.75% permethrin + 4% PBO	100	100
	Deltamethrin (0.05%) + PBO (4%)	100	100
	DDT (4%)	300	92
	Pirimiphos methyl (0.25%)	100	100
Port Victoria	Permethrin (0.75%)	217	53
	Deltamethrin (0.05%)	100	11
	Deltamethrin (0.05%) + PBO (4%)	100	96
	DDT (4%)	100	85
Bungoma	Permethrin (0.75%)	100	69
	Deltamethrin (0.05%)	100	43

N: number of mosquitoes exposed to the insecticide

**Table 4:** List of the top genes of immunity, metabolic, cuticle and olfactory

Gene ID	Symbol	Chr.	FC (R vs S)	FC (C vs S)	Resistant (read count)	Unexposed (read count)	Susceptible (read count)	Group
AFUN008117	AFUN008117	2	2.4	2.1	120.9	145.2	178.5	Cytochrome
AFUN015889	CYP6P9b	2	6.2	4.7	946.4	1423.4	1519.4	Cytochrome
AFUN015792	CYP6P9A	2	3.5	2.5	568.3	893.9	925.1	Cytochrome
AFUN010918	CYP6N1	2	3.4	3.9	341.5	306.3	726.8	Cytochrome
AFUN001383	CYP9J5	3	2.1	2	215.7	469.7	293.4	Cytochrome
AFUN015735	CYP49A1	3	3.4	2.9	160.5	113.2	218.2	Cytochrome
AFUN005715	CYP315A1	X	2.3	NS	138.6	167.1	168.2	Cytochrome
AFUN015888	CYP6P5	2	6.3	9.5	129.3	220.2	288.1	Cytochrome
AFUN020895	AFUN020895	2	6.7	5.6	394.7	604.3	506.2	Cytochrome
AFUN019365	AFUN019365	2	10.4	10.7	267.8	333.2	412.2	Cytochrome
AFUN007549	CYP9K1	X	10.5	10.5	3310.9	5502.8	4501.7	Cytochrome
AFUN015938	CYP9M1	2	2.1	NS	384.6	452.2	414.9	Cytochrome
AFUN015956	CYP304B1	2	4.5	7	261.2	109.1	373.3	Cytochrome
AFUN015957	CYP304C1	2	2.5	NS	230.7	341.1	241.2	Cytochrome
AFUN016010	GSTD1	2	3.4	3.3	4552.7	4424.7	5215.8	Glutathione s-transferase
AFUN007291	GSTT2	X	2.5	2.2	143.4	116.3	164.3	Glutathione s-transferase
AFUN011410	GSTD7	2	2.1	NS	143	128.1	214.9	Glutathione s-transferase
AFUN015767	GSTD11	2	2.7	NS	13.3	20.1	20.3	Glutathione s-transferase
AFUN015839	GSTD3	2	2.8	NS	241.9	515.5	281.2	Glutathione s-transferase
AFUN016008	GSTE6	2	9.7	6.6	99.1	122.4	106.9	Glutathione s-transferase
AFUN004194	Or42	2	2.5	NS	6	15	13.7	Odorant receptor
AFUN018482	AFUN018482	3	7.5	9.9	9.3	0	4.3	Peptidase S1 domain-containing protein
AFUN018981	AFUN018981	3	3.1	5.3	9	26.1	22	Peptidase S1 domain-containing protein
AFUN018580	AFUN018580	3	11.5	11	210.4	127.4	212.8	Peptidase S1 domain-containing protein
AFUN019220	AFUN019220	2	5.2	5.7	390.5	458.9	577.3	ABC transporter
AFUN015896	AFUN015896	2	2.1	NS	142.7	112	151.9	CLIP-domain serine protease
AFUN021427	AFUN021427	2	2.3	NS	11.6	11	8.3	Cuticular protein
AFUN021428	AFUN021428	2	2.7	NS	15.6	7	8.3	Cuticular protein
AFUN019106	AFUN019106	3	3.1	NS	8	8.7	20.4	Cuticular protein
AFUN019845	UGT302A3	3	3.2	2	421.3	681.5	634.9	UDP-glycosyltransferases
AFUN011266	UGT310B2	2	NS	3.1	16.7	16	38.6	UDP-glycosyltransferases
AFUN020198	UGT308D2	3	2	NS	22.3	46	49.1	UDP-glycosyltransferases
AFUN016302	UGT306A3	3	NS	2	279.6	318.2	391.4	UDP-glycosyltransferases
AFUN003620		2	2	2.1	320.2	325.8	437.8	UDP-glycosyltransferases
AFUN016205		3	NS	3.8	250.6	123.4	403.2	sulfotransferase
AFUN016207		3	2.1	2.4	170.8	193.5	234.9	sulfotransferase

R: resistant field mosquito population that survived the pyrethroid exposure, S: susceptible FANG colony, C: unexposed/control field mosquito population, FC: fold change, NS: not significant

## Data Availability

The RNA-Seq raw reads of the *An. funestus* data obtained were submitted to the NCBI Sequence Read Archive (SRA) under the BioSample accession number: SAMN39976597-SAMN39976605 and bioproject accession number: PRJNA1077587.

## List Of Abbreviations

ABCs: ATP-binding cassettes
COEs: carboxylesterases
CPs: cuticular proteins
CYPs: cytochrome P450s
DDT: dichlorodiphenyltrichloroethane
FC: fold change
FDR: false discovery rate
GO: gene ontology
GSTs: glutathione S-transferases
IMPs: imipenemase
ncRNAs: non-coding RNAs
OPs: olfactory proteins
PBO: piperonyl butoxide
PCA: principal component analysis
UGTs: UDP-glycosyltransferases
